# Prediction of Incidental Osteoporotic Fractures at Vertebral-Specific Level Using 3D Non-Linear Finite Element Parameters Derived from Routine Abdominal MDCT

**DOI:** 10.3390/diagnostics11020208

**Published:** 2021-01-30

**Authors:** Long Yu Yeung, Nithin Manohar Rayudu, Maximilian Löffler, Anjany Sekuboyina, Egon Burian, Nico Sollmann, Michael Dieckmeyer, Tobias Greve, Jan S. Kirschke, Karupppasamy Subburaj, Thomas Baum

**Affiliations:** 1 Engineering Product Development (EPD) Pillar, Singapore University of Technology and Design (SUTD), Singapore 487372, Singapore; longyu_yeung@mymail.sutd.edu.sg (L.Y.Y.); rayudu_nithin@mymail.sutd.edu.sg (N.M.R.); 2 Department of Diagnostic and Interventional Neuroradiology, Klinikum Rechts der Isar, School of Medicine, Technical University of Munich, Ismaninger Street 22, 81675 Munich, Germany; m.loeffler@tum.de (M.L.); anjany.sekuboyina@tu.de (A.S.); egon.burian@tum.de (E.B.); nico.sollmann@tum.de (N.S.); michael.dieckmeyer@tum.de (M.D.); tobias.greve@med.uni-muenchen.de (T.G.); jan.kirschke@tum.de (J.S.K.); thomas.baum@tum.de (T.B.); 3 TUM-Neuroimaging Center, Klinikum Rechts der Isar, Technical University of Munich, 81675 Munich, Germany; 4 Department of Diagnostic and Interventional Radiology, University Hospital Ulm, Albert-Einstein-Allee 23, 89081 Ulm, Germany; 5 Department of Neurosurgery, Ludwig-Maximilians-University, Marchioninistraße 15, 81377 Munich, Germany; 6 Changi General Hospital, 2 Simei Street 3, Singapore 529889, Singapore

**Keywords:** finite element analysis, multidetector computed tomography, osteoporosis, spine, incidental vertebral fracture

## Abstract

To investigate whether finite element (FE) analysis of the spine in routine thoracic/abdominal multi-detector computed tomography (MDCT) can predict incidental osteoporotic fractures at vertebral-specific level; Baseline routine thoracic/abdominal MDCT scans of 16 subjects (8(m), mean age: 66.1 ± 8.2 years and 8(f), mean age: 64.3 ± 9.5 years) who sustained incidental osteoporotic vertebral fractures as confirmed in follow-up MDCTs were included in the current study. Thoracic and lumbar vertebrae (T5-L5) were automatically segmented, and bone mineral density (BMD), finite element (FE)-based failure-load, and failure-displacement were determined. These values of individual vertebrae were normalized globally (g), by dividing the absolute value with the average of L1-3 and locally by dividing the absolute value with the average of T5-12 and L1-5 for thoracic and lumbar vertebrae, respectively. Mean-BMD of L1-3 was determined as reference. Receiver operating characteristics (ROC) and area under the curve (AUC) were calculated for different normalized FE (K_load_, K_displacement_,K_(load)g_, and K_(displacement)g_) and BMD (K_BMD_, and K_(BMD)g_) ratio parameter combinations for identifying incidental fractures. K_load_, K_(load)g_, K_BMD_, and K_(BMD)g_ showed significantly higher discriminative power compared to standard mean BMD of L1-3 (BMD_Standard_) (AUC = 0.67 for K_load_; 0.64 for K_(load)g_; 0.64 for K_BMD_; 0.61 for K_(BMD)g_ vs. 0.54 for BMD_Standard_). The combination of K_load_, K_displacement_, and K_BMD_ increased the AUC further up to 0.77 (*p* < 0.001). The combination of FE with BMD measurements derived from routine thoracic/abdominal MDCT allowed an improved prediction of incidental fractures at vertebral-specific level.

## 1. Introduction

Osteoporosis is a skeletal disease which results in fragility fractures due to loss of bone matrix and deterioration of the microarchitecture and structural integrity of bone tissue [1,2,3,4]. It is estimated to have caused ~3.3 million fragility fractures in 2030, in the EU alone [5]. Considering the world population is aging, the economic and social burden of fracture related disabilities and healthcare is significant [6]. Vertebral compression fractures (VCF’s) are the most common type of osteoporotic fractures. In the elderly population, the VCF’s generally results in bone failure [7]. Studies have shown that patients who suffered from osteoporotic vertebral fractures may not be able to return to the pre-fracture physical health as well as the quality of life [5,6]. Thus, it is vital to identify patients with high fracture risk at earlier stages for a timely initiation of therapy. The spine is one of the clinically most relevant anatomical locations in osteoporosis, since after hip fractures the second highest number of osteoporotic fractures occur at the spine [5]. The thoracic and lumbar are two important sections of the spine. Anatomically thoracic and lumbar sections can further be divided into thoracic (T1-T9), thoracolumbar junction (T10-L2), and lumbar (L3-L5) sections. Thoracic vertebrae support the ribcage and are functionally rigid, whereas the lumbar spine is flexible and transfer majority of the human body weight. Thoracic and lumbar vertebrae are major fracture prone regions at the spine. In North America, about 50–60% of the thoracolumbar fractures effects the thoracolumbar junction, 10–14% occurs in lower lumbar spine and 25–40% fractures occur in thoracic spine [8]. 

For diagnosing osteoporosis and assessing fracture risk, bone mineral density (BMD) values are measured. Dual-energy X-ray absorptiometry (DXA)-based aerial bone mineral density (aBMD) bone measures and corresponding T- and Z-scores are considered as the standard parameters for diagnosis of osteoporosis [9,10]. The opportunistic assessment of BMD derived from multi-detector computed tomography (MDCT) scans acquired for other clinical purposes can provide valuable information for osteoporosis assessment [11,12]; this goes hand in hand with reducing the amount of radiation dose and costs [13]. Specifically, BMD values of the lumbar spine derived from routinely acquired contrast-enhanced MDCT data can differentiate patients with no, existing and incidental osteoporotic vertebral fractures [14]. 

However, studies have shown that the BMD alone is not sufficient for measuring the bone strength and health [13,15]. For identification of fracture risk, it is important to consider other proven risk factors along with BMD for accurate assessment [16]. Bone is a complex nonhomogeneous structure and interactions between the different structural elements need to be considered for accurate prediction of fracture risk.

Finite element (FE) analysis is a computational approach which is used to solve biomechanical problems, like bone strength calculations [17,18,19,20,21]. In FE analysis, patient-specific three-dimensional (3D) tissue models are generated from the MDCT image data. Then, the image intensity (Hounsfield units (HU))-based material properties are assigned to the FE mesh and realistic loading and boundary conditions are applied to calculate the biomechanical response [17,22,23,24,25]. 

FE results can be used to predict the vertebral fracture risk. Studies have shown that the FE vertebral failure load has higher fracture discrimination power compared to BMD for fracture risk prediction [26,27,28]. Allaire et. al. have demonstrated that FE derived vertebral bone strength is able to better predict incidental vertebral fractures compared to CT-based BMD [17]. Kopperdahl et. reported that the combination of the FE derived femoral bone strength with aBMD values increased the fracture classification compared to individual values [25]. While these studies demonstrated a certain preponderance of FE based analysis, most studies mainly investigated single vertebrae only. This is an important limitation, since it is well known that the spine has an inhomogeneous BMD distribution and that BMD loss is dependent on the vertebral level [29]. Furthermore, most studies used dedicated MDCT scans for FE analysis. 

Thus, the scope of the current work was to investigate whether FE and BMD measures and their combinations derived from the routine thoracic and abdominal MDCT can predict incidental osteoporotic fracture at vertebral-specific level. 

## 2. Materials and Methods

### 2.1. Subjects

A cohort of 16 subjects (eight male, mean age: 66.1 ± 8.2 years and eight female, mean age: 64.3 ± 9.5 years) was included in the current study. These subjects were retrospectively identified in our institution’s digital picture archiving and communication system (PACS). They had a history of cancer (such as esophageal, lung, or colorectal cancer) and chemotherapy. They underwent thoracic and abdominal MDCT exams as long-term follow-up to rule out tumor recurrence. All included subjects were Caucasian, while no patients with different ethnicity matched the inclusion/exclusion criteria. 

All subjects with a known history of bone diseases, including hematologic, metastatic, and metabolic disorders, aside from osteoporosis, were excluded. This was done by checking all available image data and the electronic medical records of each subject. The patients with history of cancer with no distant metastases were curatively treated and underwent MDCT imaging to rule out tumor recurrence. Only subjects with an incidental osteoporotic vertebral fracture at the time of the follow-up MDCT were selected. Fracture status was assessed by a board-certified radiologist in the sagittal reformations of the spine using the Genant classification system [30]. All patients had a history of chemotherapy, but no relevant comorbidities affecting the bone metabolism. The mean Body Mass Index (BMI) of the included patients was 23.1 ± 3.2 m/kg^2^. The overall inclusion and exclusion criteria followed in the current study are shown in Table 1. Baseline MDCT exams had to be performed at the same single MDCT scanner with a specific protocol as outlined below for quality assurance of the quantitative MDCT data. The local institutional review board approved the current study, and the requirement of the written consent was waived due to the retrospective nature of the study. 

**Figure 1:**
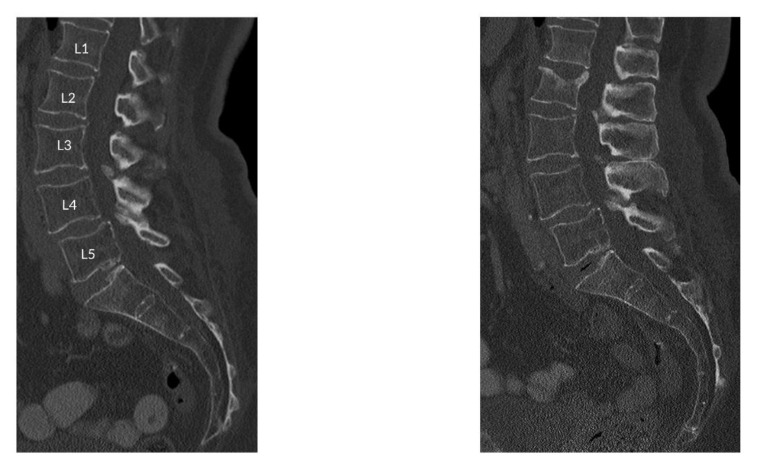
Sagittal reformation of a representative patient with incidental osteoporotic vertebral fracture of L2 at follow-up.

The incidental fractured vertebral levels and follow-up scan duration were as following: In 11 subjects, one incidental fracture was observed at T7 (16 months), T8 (37 months), T9 (25 months), T11 (10 months), T12 (18 months), T12 (23 months), L1 (5 months), L1 (10 months), L2 (10 months), L3 (17 months), and L5 (35 months), vertebral levels, respectively. In three subjects, two incident fractures were observed at T5 and T12 (14 months), L1 and L2 (16 months), T5 and T9 (41 months), vertebral levels, respectively. Finally, three incidental fractures were observed in two subjects at T12, L1, L2 (28 months) and T10, T11, T12 (26 months) vertebral levels. Figure 1 shows the sagittal reformation of a representative patient with incidental osteoporotic vertebral fracture of L2 at follow-up. The sagittal reformation of baseline and follow-up scan MDCT images for all the patients are shown in the Supplementary Figure S1.

### 2.2. Image Acquisition

All routine abdominal contrast-enhanced MDCT scans were performed with the same 64-row MDCT scanner (Somatom Sensation Cardiac 64; Siemens Medical Solutions, Erlangen, Bavaria, Germany). Scanning parameters were 120 kVp tube voltage, adapted tube load of averaged 200 mAs, and minimum collimation of 0.6 mm. Sagittal reformations of the spine were reconstructed with a slice thickness of 3 mm with a standard bone kernel of the manufacturer. Examinations were performed after standardized administration of intravenous contrast medium (Imeron 400; Bracco, Konstanz, Germany) using a high-pressure injector (Fresenius Pilot C; Fresenius Kabi, Bad Homburg, Germany). Intravenous contrast medium injection was performed with a delay of 70 s, a flow rate of 3 mL/s, and a body weight–dependent dose (80 mL for body weight up to 80 kg, 90 mL for body weight up to 100 kg, and 100 mL for body weight over 100 kg). Additionally, all patients were given 1000 mL oral contrast medium (Barilux Scan; Sanochemia Diagnostics, Neuss, Germany). A reference phantom (Osteo Phantom, Siemens Medical Solutions, Erlangen, Bavaria, Germany) was placed in the scanner mat beneath the patients in all MDCT scans. All single vertebrae with prevalent fracture at baseline were excluded from the further analysis. Fracture status was assessed by a board-certified radiologist in the sagittal reformations of the spine using the Genant classification system.

### 2.3. MDCT-Derived BMD Calculation

For BMD measurements from T5-L5, the most central slice depicting the vertebral body was selected. Regions of interest (ROIs) were manually placed equidistant to both endplates in the trabecular compartment of the anterior vertebral body, for each vertebra (T5-L5) and patient. HU values of the ROIs were extracted. Using the Siemens Osteophantom with two phases: water (HA_w_) and bone (HA_b_) with values of 0 and 200 mg/mL hydroxyapatite (HA), respectively, the BMD values were calculated based on the following relation: (BMD)_MDCT_ = [HA_b_/(HU_b_ − HU_w_)] × (HU − HU_w_) [31]. HU_w_ and HU_b_ represents the intensity values of water and bone like phantoms. The MDCT derived BMD values were converted to standard QCT BMD values using the linear relation (BMD)_QCT_ = 0.69 × (BMD)_MDCT_ − 11 mg/mL [12].

### 2.4. Finite Element Modelling and ANALYSIS

**Figure 2:**
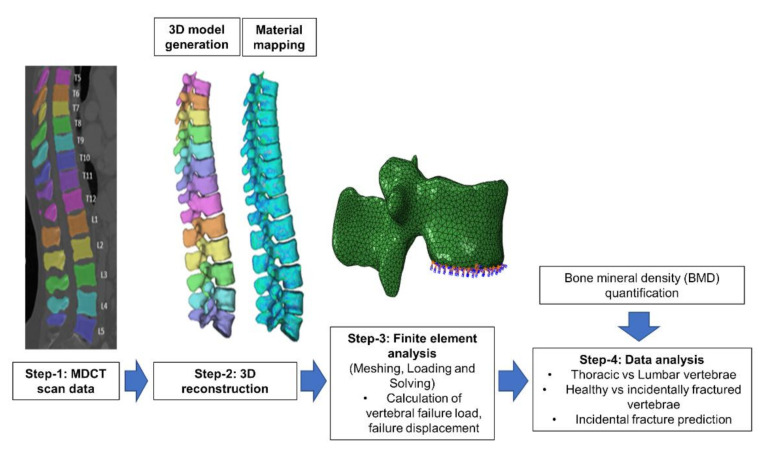
Modelling and analysis methodology followed in the current computational study for identifying incidental fractures.

Figure 2 shows the stepwise analysis methodology followed in the current computational study. In step 1 routine MDCT scan data is selected retrospectively and the spine was automatically segmented. In step 2, patient-specific 3D model has been generated and this model was then meshed using tetrahedral elements, image intensity based non-linear material properties were applied to the FE mesh. In step 3, compression loading condition was applied on the vertebrae and nonlinear FE analysis was performed using a commercial software Abaqus (version 6.10, Hibbitt, Karlsson, and Sorensen, Pawtucket, RI, USA) to calculate the FE failure load, and displacement [22,32]. In step 3, in addition to FE based results, we have also calculated the BMD values from MDCT images [33]. Lastly, in step 4 the obtained parameters were analyzed to identify those parameters and their combinations that predict incidental vertebral fracture best.

#### 2.4.1. Image Segmentation and Meshing

In this study, considering the Biomechanical importance of both weight-bearing regions of the vertebral bone i.e., body and posterior elements, vertebrae T5 to L5 including the posterior elements were automatically segmented from the MDCT images using a deep learning-driven framework (https://anduin.bonescreen.de) [34]. This algorithm is freely usable and fully-automated and identifies the spine, labels each vertebral body, and creates corresponding segmentation masks. 

#### 2.4.2. 3D Reconstruction and Meshing

The MDCT scan data along with the segmentation masks of T5 to L5 were imported to the commercial 3D medical image processing software Mimics (Materialise NV, Harislee, Belgium) and 3D vertebral models were generated. These 3D models were then imported to the 3-Matic software (Materialise NV, Harislee, Belgium) for meshing. To capture the bone contour accurately, tetrahedral element (C3D4 in Abaqus material library) was used for FE meshing. The nonhomogeneous and non-isotropic material behavior of the bone was captured by considering image intensity (Hounsfield unit (HU))-based material mapping relation [18,35,36,37,38,39]. Table 2 shows the HU-density-elasticity material mapping relations used in the current study [40,41,42]. The cortical bone was simplified and assumed as denser trabecular bone and same material mapping relations are used for both the regions [28,35].

Mesh sensitivity study was performed to maintain the accuracy of the FE model. In this study, we varied the maximum element edge length from 1.5 to 3 mm [22,32] with an increment of 0.25 mm and identified that 2 mm was giving the mesh independent results and the same size was used for all vertebral models.

#### 2.4.3. Finite Element Analysis 

**Figure 3:**
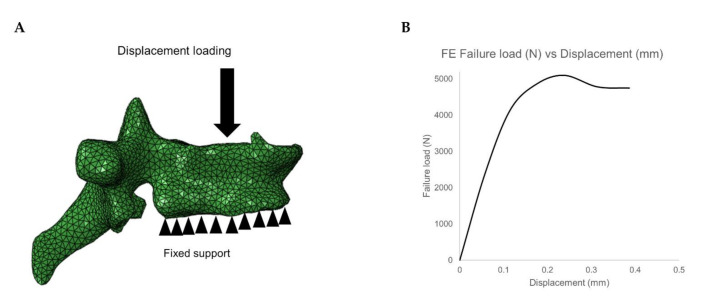
(**A**) Loading and boundary condition on the vertebrae. (**B**) Failure load and displacement variation for the vertebrae under compressive loading condition.

The meshed and material mapped 3D vertebrae model was then imported into the commercial analysis software ABAQUS version 6.10 for downstream FE analysis, which includes loading and boundary conditions application and solving the model. In the current study, compression loading condition was simulated by constraining all the nodes on the inferior surface of the vertebrae and displacement loading is applied on the superior surface of the vertebrae (cf. Figure 3A). Then the FE model is solved, and vertebral failure load of T5-L5 is calculated. The peak of the load-displacement curve is considered as failure load and the corresponding displacement for the failure load is considered as failure displacement (cf. Figure 3B). The FE methodology used in the current study has been validated experimentally in the previous studies [22,23,28,39,43,44].

### 2.5. Statistical Analysis

The data analysis was performed using Microsoft Excel (version 16.27 (2019); Microsoft Corporation, Redmond, WA, USA) and IBM SPSS Statistics for Windows (version 25.0; IBM Corp., Armonk, NY, USA). For all statistical tests, a two-sided level of significance of 0.05 was considered. Normalized ratios (K) were calculated globally (*K_(x)g_*) by dividing the absolute value of FE and BMD results with the average of the same parameter for L1-3 (as standard of reference) and locally (*K_x_*) for thoracic and lumbar vertebrae for all the FE and BMD results by dividing the individual values at each vertebral level with the average of the same parameter for the thoracic (T5-12) and lumbar (L1-5) region, respectively. All the FE and BMD results were calculated for the baseline MDCT data:

$$Global\;Normalized\;ratio\;\left(K_{\left(x\right)g}\right)=\frac{absolute\;value\;of\;FE\;and\;BMD\;results\;}{{\left(L1-3\right)}_{average}}$$

$$Local\;Normalized\;ratio\;\left(K_{x}\right)=\frac{absolute\;value\;of\;FE\;and\;BMD\;results\;}{{\left(T5-12\right)}_{average}\;or\;{\left(L1-5\right)}_{average}}$$

where *x* is the considered variable.

These normalized values are used for comparing the healthy (H; i.e., non-fractured vertebrae at baseline as well as follow-up) and incidentally fractured vertebrae (F). 

The Kolmogorov-Smirnov test indicated non-normally distributed data for the majority of parameters. Therefore, Mann-Whitney U tests were performed to identify the statistically significant parameters that differentiate H and F cases. Descriptive statistics including mean ± standard deviation (SD) were reported for all the ratio values. Mean BMD of L1-3 was determined as reference standard. For identifying the best parameter combination for predicting incidental fractures, receiver operating characteristics (ROC) curve analyses were plotted and the area under the curve (AUC) was determined.

## 3. Results

### 3.1. Comparison of FE and BMD Parameters for Thoracic and Lumbar Region

**Figure 4:**
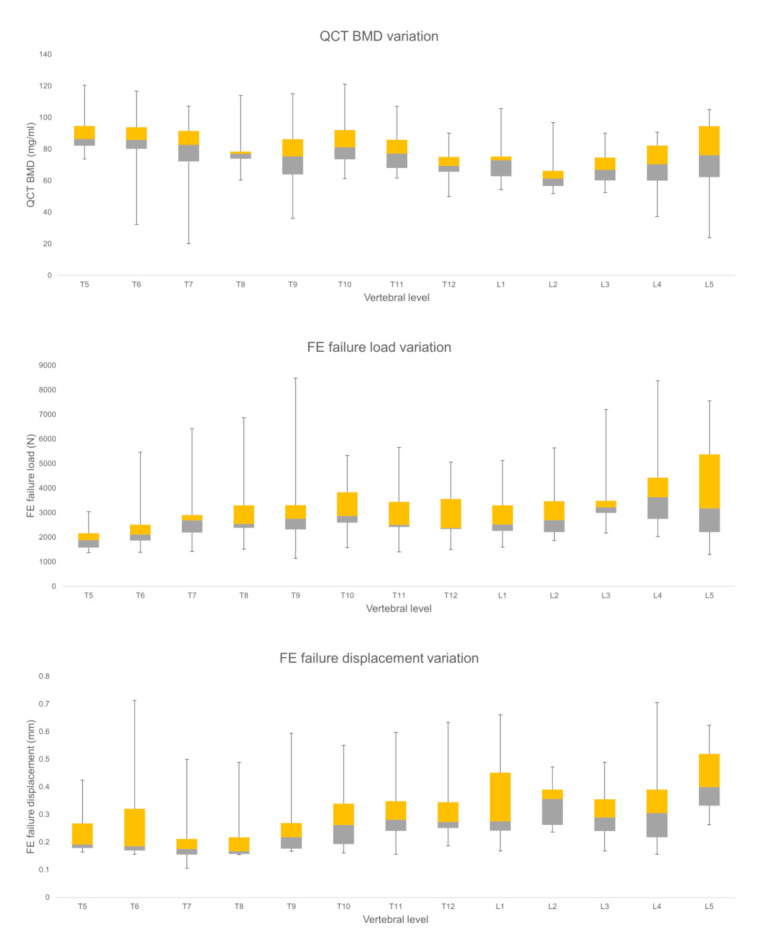
Finite element (FE), and Qct-BMD parameters variation for each vertebral level for healthy vertebrae (Median, quartile 2, 3, with the total range are shown).

The mean FE failure load values for the thoracic vertebrae amounted to 2768.58 ± 1173.06 N and for lumbar vertebrae 3500.81 ± 1540.06 N, respectively. The mean FE failure displacement values for thoracic vertebrae was 0.26 ± 0.12 mm and for lumbar vertebrae 0.35 ± 0.13 mm, respectively. The mean BMD values for thoracic vertebrae amounted to 80.47 ± 17.56 mg/mL and for lumbar vertebrae 69.77 ± 17.01 mg/mL, respectively. FE failure load, FE failure displacement, and BMD were significantly (*p* < 0.05) different between the thoracic and lumbar spine regions. Figure 4 shows the variation of the considered parameters at all the vertebral levels (T5-L5).

### 3.2. Comparison of FE and BMD Parameters for Healthy and Incidentally Fractured Vertebrae

When global ratio values were considered, the mean FE failure load ratio values for the healthy vertebrae was 1.00 ± 0.31 and for incidentally fractured vertebra 0.85 ± 0.18, respectively. The mean FE failure displacement ratio values for healthy vertebrae was 0.92 ± 0.36, whereas incidental fractured vertebrae had a FE failure displacement ratio of 1.12 ± 0.62. The mean BMD ratio values for healthy vertebrae was 1.14 ± 0.27 and for incidental fractured vertebrae 1.03 ± 0.19 respectively (Table 3). K_(load)g_, and K_(displacement)g_, and K_(BMD)g_ were significantly different between healthy and fractured vertebrae (*p* < 0.05).

When local ratio values are considered, the mean FE failure load ratio values for the healthy vertebrae was 1.01 ± 0.22 and for incidentally fractured vertebra 0.87 ± 0.19, respectively. The mean FE failure displacement ratio values for healthy vertebrae was 0.98 ± 0.32, whereas incidental fractured vertebrae had a FE failure displacement ratio of 1.18 ± 0.47. The mean BMD ratio values for healthy vertebrae was 1.01 ± 0.16 and for incidental fractured vertebrae 0.92 ± 0.13, respectively (Table 3). K_BMD_, K_displacement_, and K_load_ values were able to significantly differentiate healthy and fractured vertebrae (*p* < 0.05). The mean BMD values of L1-3 (BMD_Standard_) for healthy vertebrae was 68 ± 13 mg/mL and for incidentally fractured vertebrae 68 ± 13 mg/mL, respectively.

### 3.3. Incidental Fracture Prediction Using Different FE and BMD Parameter Combination

**Figure 5:**
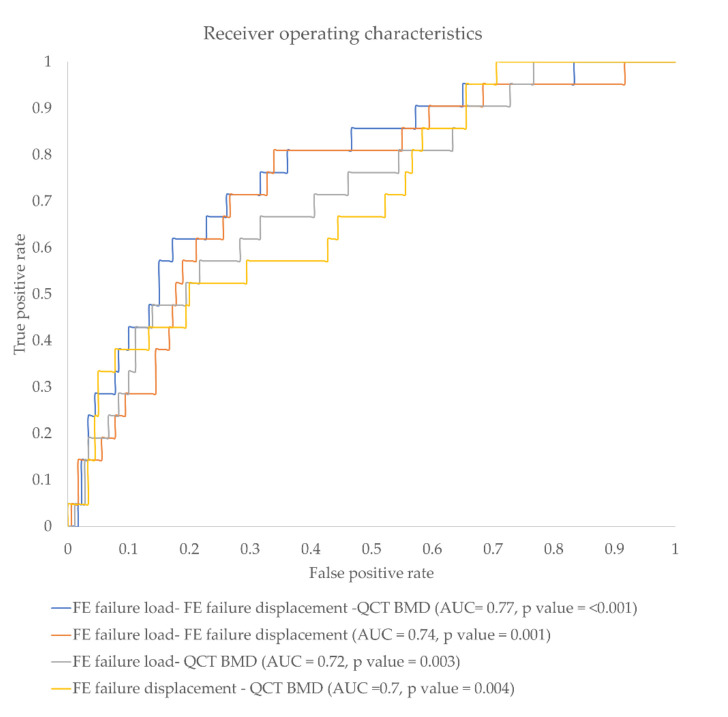
Receiver operating characteristics (ROC) curve showing false-positive rate vs. true-positive rate for different parameter combinations.

K_load_, K_(load)g_, K_BMD_, K_(BMD)g_, and K_displacement_ showed significantly higher discriminative power compared to standard mean BMD of L1-3 (BMD_Standard_) (AUC = 0.67, *p* =0.005 for K_load_; AUC = 0.64, *p* = 0.021 for K_(load)g_; AUC = 0.64, *p* = 0.025 for K_BMD_; AUC = 0.61, *p* = 0.062 for K_(BMD)g_; AUC = 0.61, *p* = 0.017 for K_displacement_ vs. 0.54, *p* = 0.976 for BMD_Standard_). K_(displacement)g_ showed an AUC of 0.56 (*p* = 0.04). When all global parameters were combined the discrimination power significantly increased to AUC = 0.71, *p* = 0.011. When the local parameters were combined, incidental fracture discrimination power significantly increased further up to AUC = 0.77 (*p* < 0.001) (Table 4). Figure 5 shows the ROC curves and corresponding AUC values for combined local ratio parameters.

## 4. Discussion

In the current work, we investigated the performance of different parameters in predicting incidental osteoporotic fractures at vertebral specific level. The parameter combination of K_load_, K_displacement_, and K_BMD_ was able to predict incidental vertebral fractures with high discrimination power (AUC = 0.77). 

The size, shape, and load bearing capacity of the spine is regionally different. It is important to understand the load bearing capacity of each region for assessment of fracture risk. In the current work, we tried to understand the variation of FE results in the lumbar and thoracic regions. We observed that the FE parameters (i.e., failure load and failure displacement) showed a significant difference between the thoracic and lumbar regions. The size of the lumbar vertebrae is higher in comparison to thoracic vertebrae, and the lumbar vertebrae are subjected to higher stress under different loading conditions [45]. This can be the reason for the observed higher values for FE failure load and FE failure displacement values in the lumbar region. Anitha et. al. have shown that the mean failure load for the thoracic region is lower compared to the lumbar region [28]. Kang et. al. reported that the lumbar vertebral body diameter is significantly higher compared to the thoracic vertebral body [46]. Thus, regional variations have to be taken into account for vertebral-specific fracture risk prediction based on FE analysis.

The K_load_, K_(load)g_, K_BMD_ and K_(BMD)g_ values observed were higher for H compared to F cases, and, furthermore, the K_displacement_ and K_(displacement)g_ ratios were higher for F as compared to H cases. Before fracturing, any bone will undergo changes like bone deterioration, and strength reduction. As affected vertebrae are going to fail in the future, they show higher K_displacement_, K_(displacement)g_ values as well as lower K_load_, K_(load)g_, K_BMD_ and K_(BMD)g_ values for F cases. Of note, Chandran et. al. have observed a similar trend as due to osteoporosis the bone is weakened and failure load is reduced significantly [40]. 

In this study, we tried to identify the best FE- and QCT-based BMD parameter combinations for identifying an incidental vertebral fracture. We observed that K_load_, K_BMD_, K_(load)g_, K_(BMD)g_ and K_displacement_ showed significantly higher discriminative power compared to K_(displacement)g_ and absolute mean BMD of L1-3 (BMD_Standard_) (AUC = 0.67 for K_load_; 0.64 for K_(load)g_; 0.64 for K_BMD_; 0.61 for K_(BMD)g_; 0.61 for K_displacement_ vs. 0.54 for BMD_Standard_). Currently, QCT-based volumetric BMD (vBMD) values averaged over L1-3 are used as reference standard in clinical routine. Studies have shown that BMD values alone are not able to predict the occurrence of a fracture accurately [47]. Imai et al. have shown that compared to vBMD values (AUC = 0.767), FE-based vertebral bone strength (AUC = 0.822) is superior in fracture prediction [26]. Allaire et. al. have shown that vertebral bone strength (AUC = 0.804) is able to predict the fracture risk accurately compared to CT-based BMD values (AUC = 0.715) [17]. The major drawback of the BMD measures is that these do not consider the bone quality factors like bone shape, morphology, critical locations, and bone mass distribution. 

The computational algorithms like FE methods are able to reconstruct the patient 3D models and capture the heterogeneous nature of bone accurately [28,37,48,49]. We have also observed that the effectiveness of the combined parameters for incidental fracture prediction is higher compared to individual analysis. Specifically, when K_BMD_ values were combined with other parameters like K_load_ and K_displacement_, the effectiveness of the future fracture prediction increased significantly AUC = 0.72 and 0.70, respectively. Finally, when all the three parameters were combined, a further increase in the fracture discriminative power was observed (AUC = 0.77). These AUC values are higher than those reported by Muehlematter et al. (AUC = 0.64) [50]. They performed a prediction of incidental fractures at vertebral-specific level by using texture analysis and machine learning algorithms. Thus, our findings suggest a better performance of FE parameters for predicting incidental vertebral fractures. We also observed that compared to the globally evaluated values, local ratio values showed a higher discrimination power (AUC = 0.71, for global vs. 0.77, for local ratio values). Using these local ratio values the clinician can identify critical vertebrae in advance. This may allow an improved fracture risk prediction and an accurate and timely treatment initiation. We have also observed that by adding FE parameters like failure displacement and failure load to the BMD values, accurately predicting the incidental fractures is possible. Using this methodology, the clinician can start the treatment well in advance and improve the efficiency of the drug treatment. 

Some limitations of the study need to be acknowledged. First, the considered cohort size for the current computational study is rather small. This is due to inclusion of patients with baseline MDCT exams from the very same MDCT scanner with a specific protocol, which increased robustness of the MDCT data. Second, in this study, the vertebrae were simulated under compression loading; however, when the model is simulated for other loading configurations like flexion, bending, or twisting, for instance, the FE results can vary accordingly. Third, a randomized control trial with a higher sample is needed before employing this method in a clinical setting. Fourth, for identifying the critical vertebrae, complete CT scans of the spine are needed. Fifth, we did not consider the physiological differences between thoracic and lumbar vertebrae and adopted a similar modeling and analysis methodology to study these sections of the spine using finite element analysis. 

## 5. Conclusions

In conclusion, the combination of FE along with BMD values derived from routine thoracic/abdominal MDCT allowed an improved prediction of incidental fractures at vertebral-specific level.

## Figures and Tables

**Table 1:** Inclusion and exclusion criteria followed in the current study for selecting the MDCT images.

Inclusion Criteria	Exclusion Criteria
Baseline thoracic and abdominal MDCT exams at the same single MDCT scanner with a specific protocol	History of bone diseases, including hematologic and metabolic disorders, aside from osteoporosis
Follow-up thoracic and abdominal MDCT exams with incidental osteoporotic vertebral fracture	Bone metastases
Availability of sagittal image reformations of the spine at baseline and follow-up MDCT	

**Table 2:** Density (ρ)—intensity (HU)—modulus (E) material mapping relations used in the current computational study for modelling the non-homogenous material behavior.

Material Properties	Unit	Mapping Relations
Apparent density (ρ_app_) [35]	Kg/m^3^	ρ_app_ = 47 + 1.122 × HU HU—Hounsfield unit
Ash density (ρ_ash_) [36]	Kg/m^3^	ρ_ash_ = 0.6 × ρ_app_
Elastic modulus (E) [35]	MPa	E_z_ = −349 + 5.82 × ρ_app_ E_x_ = E_y_ = 0.333 E_z_ Z—axial direction of the vertebra
Shear modulus (G) [18]	MPa	G_xy_ = 0.121 E_z_ G_xz_ = G_yz_ = 0.157 E_z_
Poisson ratio (V) [18]	Constant	Vxy = 0.381 Vxy = Vyz = 0.104
Maximum principal stress limit (σ) [38]	MPa	σ = 137 × ρ_ash_^1.88^, ρ_ash_ < 0.317 σ = 114 × ρ_ash_^1.72^, ρ_ash_ > 0.317
Plastic strain (ε_AB_) [37]	No unit	ε_AB_ = −0.00315 + 0.0728 ρ_ash_
Minimum principal stress limit (σ_min_) [37]	MPa	σ_min_ = 65.1 × ρ_ash_^1.93^

**Table 3:** Mean and standard deviation (sd) of bone mineral density (BMD) and finite element (FE)-based failure load and displacement normalized ratios for healthy and incidental fractured vertebrae.

**Normalized Ratio Parameter (Local)**	**Healthy** **(Mean ± sd)**	**Fractured** **(Mean ± sd)**	***p*-Value**
FE Failure load	1.01 ± 0.22	0.87 ± 0.19	0.005 *
FE Failure displacement	0.98 ± 0.32	1.18 ± 0.47	0.045 *
BMD	1.01 ± 0.16	0.92 ± 0.13	0.037 *
**Normalized Ratio Parameter (Global)**	**Healthy** **(Mean ± sd)**	**Fractured** **(Mean ± sd)**	***p*-Value**
FE Failure load	1.00 ± 0.31	0.85 ± 0.18	0.003 *
FE Failure displacement	0.92 ± 0.36	1.12 ± 0.62	0.171
BMD	1.14 ± 0.27	1.03 ± 0.19	0.054

* *p*-value is less than the level of significance (*p* < 0.05).

**Table 4:** Area under the curve (AUC) of different finite element (FE) and bone mineral density (BMD) ratio parameter combinations for identifying incidentally fractured vertebrae.

Normalized Ratio Parameter Combination	*p*-Value	AUC
FE failure load ratio and FE failure displacement ratio	<0.001 *	0.74
FE failure load ratio and Qct BMD ratio	0.003 *	0.72
Qct BMD and FE failure displacement ratio	0.004 *	0.70
FE failure load ratio, FE failure displacement ratio, and Qct BMD ratio	<0.001 *	0.77

* *p*-value is less than the level of significance (*p* < 0.05).

## Data Availability

The raw data supporting the conclusions of this article will be made available by the authors, without undue reservation.

## Supplementary Materials

The following are available online at https://www.mdpi.com/2075-4418/11/2/208/s1, Figure S1: Sagittal reformation of baseline (left) and follow-up (right) MDCT showing the incidental osteoporotic vertebral fractures at follow-up.

## Abbreviations

3D	Three-dimensional
aBMD	Areal bone mineral density
AUC	Area under the curve
BMD	Bone mineral density
CT	Computed tomography
DXA	Dual-energy X-ray absorptiometry
F	Incidentally fractured vertebrae
FE	Finite elements
H	Healthy vertebrae
HU	Hounsfield Units
K	Normalized ratio parameter
K_displacement_	Normalized FE failure displacement ratio
K_load_	Normalized FE failure load ratio
K_BMD_	Normalized BMD ratio
K_(displacement)g_	Normalized global FE failure displacement ratio
K_(load)g_	Normalized global FE failure load ratio
K_(BMD)g_	Normalized global BMD ratio
L	Lumbar region
MDCT	Multi detector computed tomography
PACS	Picture archiving and communication system
QCT	Quantitative computed tomography
ROC	Receiver operating characteristic
ROI	Region of interest
SD	Standard deviation
T	Thoracic region
