# Supplementary material for: Prediction of Incidental Osteoporotic Fractures at Vertebral-Specific Level Using 3D Non-Linear Finite Element Parameters Derived from Routine Abdominal MDCT
Source: Diagnostics (Basel). 2021 Jan 30;11(2):208. doi: 10.3390/diagnostics11020208 (PMC7911185; doi:10.3390/diagnostics11020208)
Supplement: Supplementary file 1 [file diagnostics-11-00208-s001.pptx]

## Slide 1
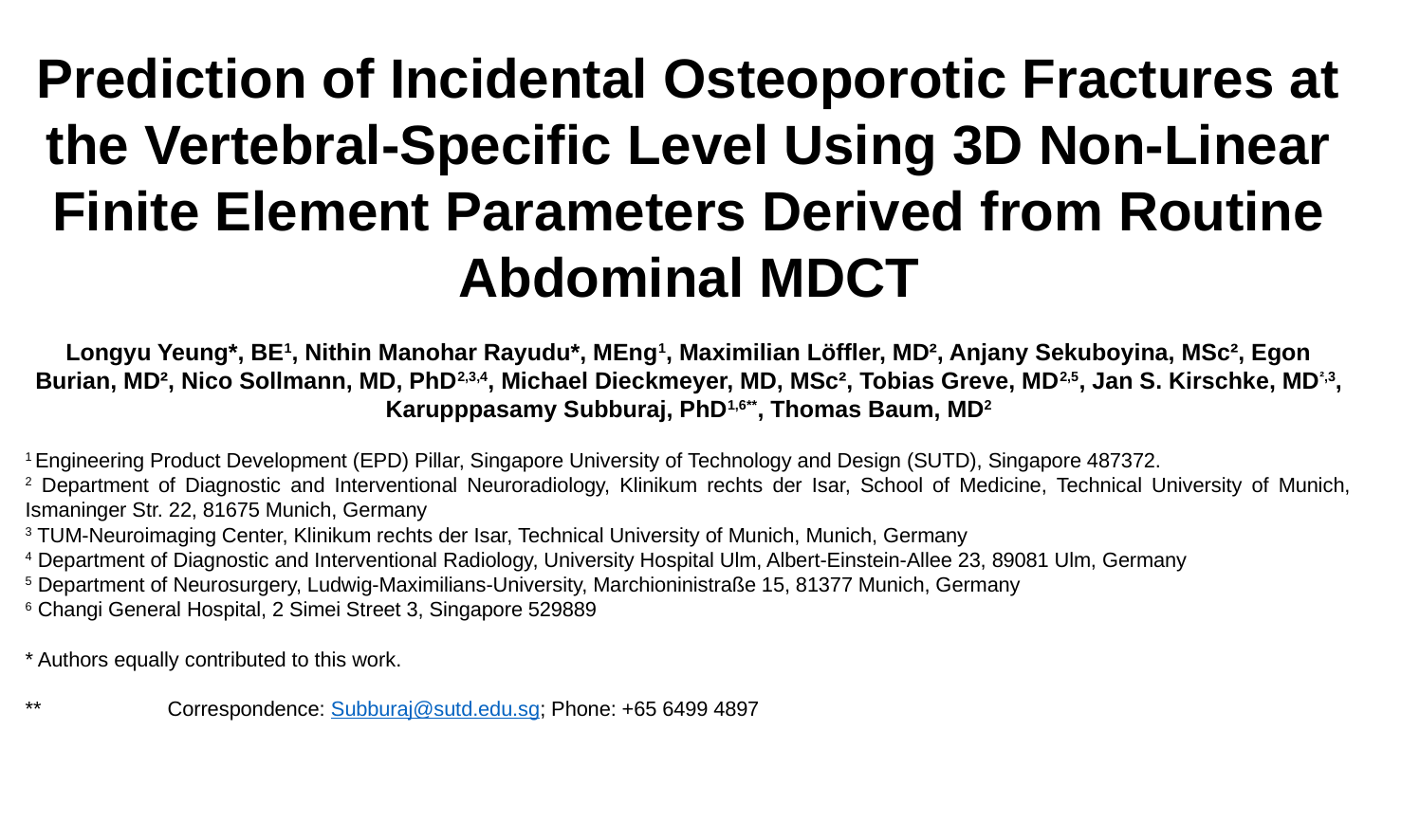

Prediction of Incidental Osteoporotic Fractures at the Vertebral-Specific Level Using 3D Non-Linear Finite Element Parameters Derived from Routine Abdominal MDCT
Longyu Yeung*, BE1, Nithin Manohar Rayudu*, MEng1, Maximilian Löffler, MD², Anjany Sekuboyina, MSc², Egon Burian, MD², Nico Sollmann, MD, PhD2,3,4, Michael Dieckmeyer, MD, MSc², Tobias Greve, MD2,5, Jan S. Kirschke, MD²,3, Karupppasamy Subburaj, PhD1,6**, Thomas Baum, MD2
1 Engineering Product Development (EPD) Pillar, Singapore University of Technology and Design (SUTD), Singapore 487372.
2 Department of Diagnostic and Interventional Neuroradiology, Klinikum rechts der Isar, School of Medicine, Technical University of Munich, Ismaninger Str. 22, 81675 Munich, Germany
3 TUM-Neuroimaging Center, Klinikum rechts der Isar, Technical University of Munich, Munich, Germany
4 Department of Diagnostic and Interventional Radiology, University Hospital Ulm, Albert-Einstein-Allee 23, 89081 Ulm, Germany
5 Department of Neurosurgery, Ludwig-Maximilians-University, Marchioninistraße 15, 81377 Munich, Germany
6 Changi General Hospital, 2 Simei Street 3, Singapore 529889
* Authors equally contributed to this work.
**	Correspondence: Subburaj@sutd.edu.sg; Phone: +65 6499 4897

## Slide 2
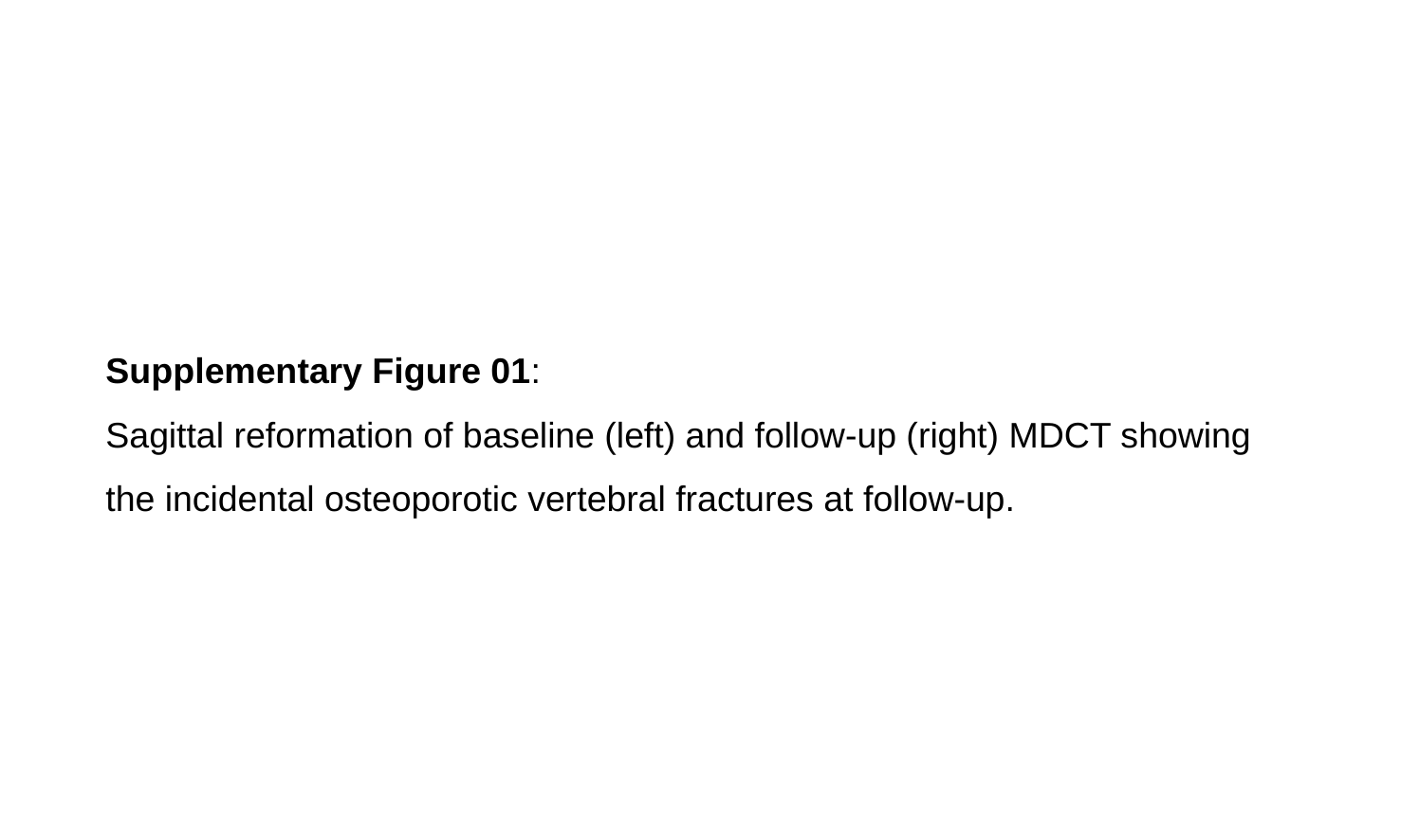

Supplementary Figure 01:
Sagittal reformation of baseline (left) and follow-up (right) MDCT showing the incidental osteoporotic vertebral fractures at follow-up.

## Slide 3
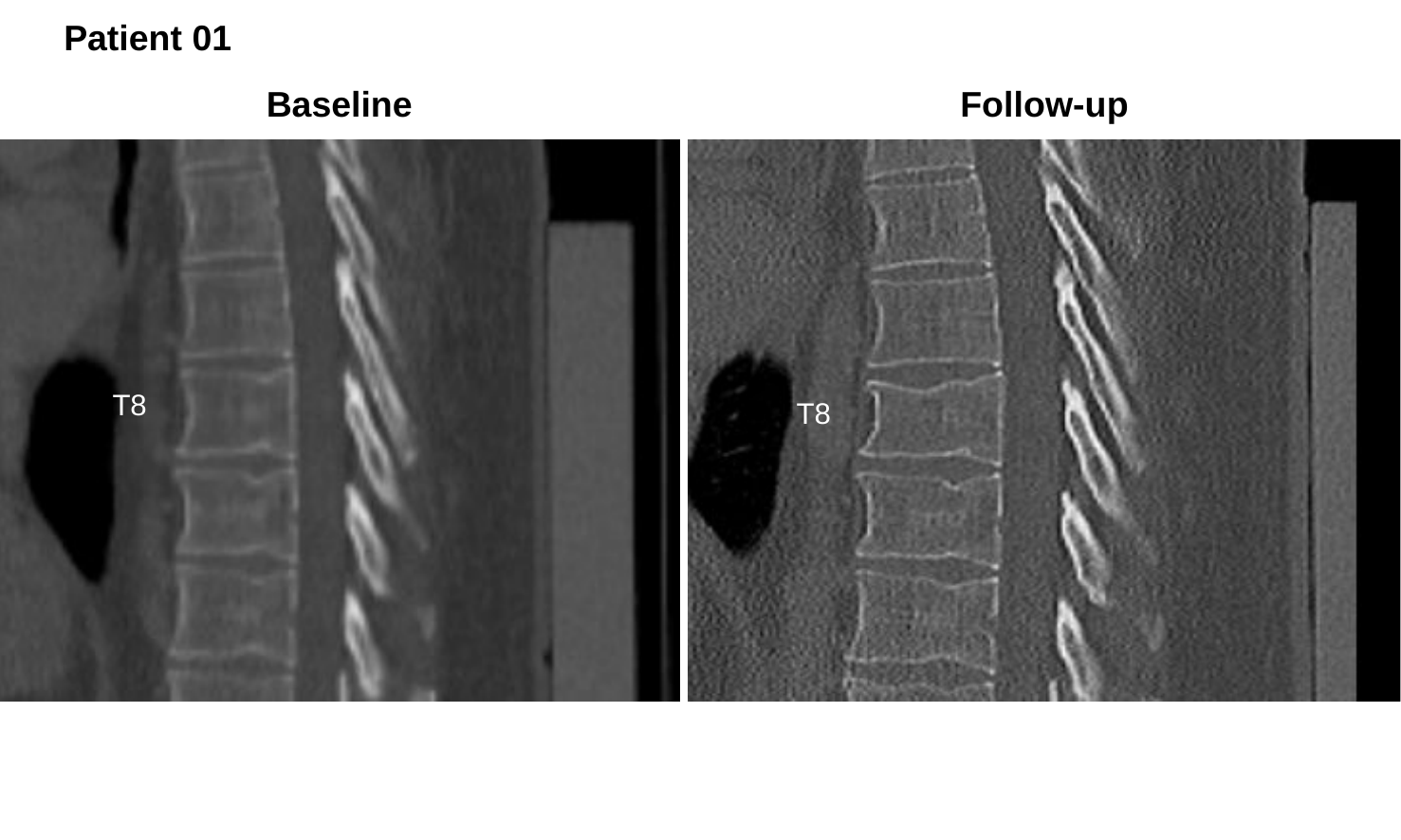

Patient 01
Baseline
Follow-up
T8
T8

## Slide 4
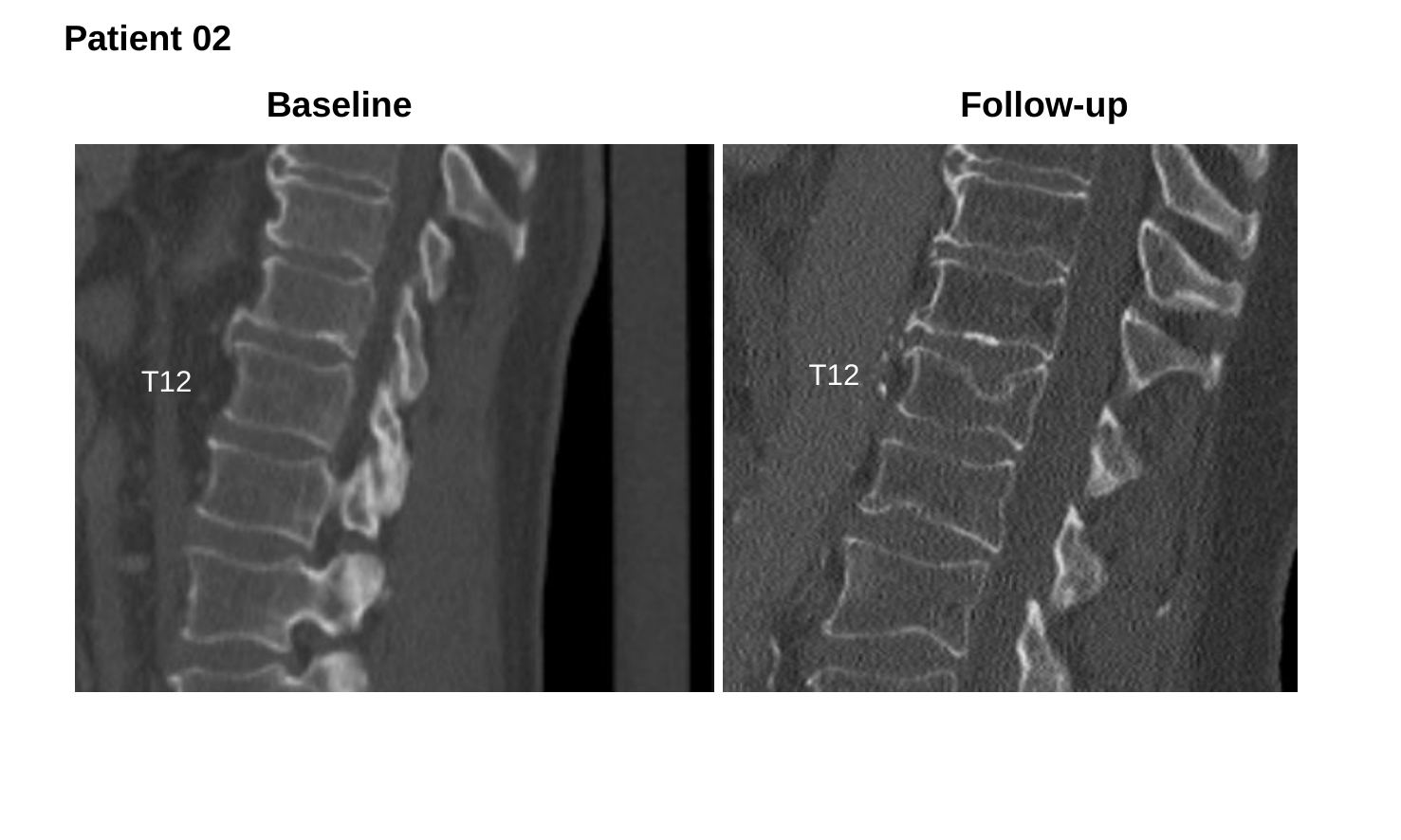

Patient 02
Baseline
Follow-up
T12
T12

## Slide 5
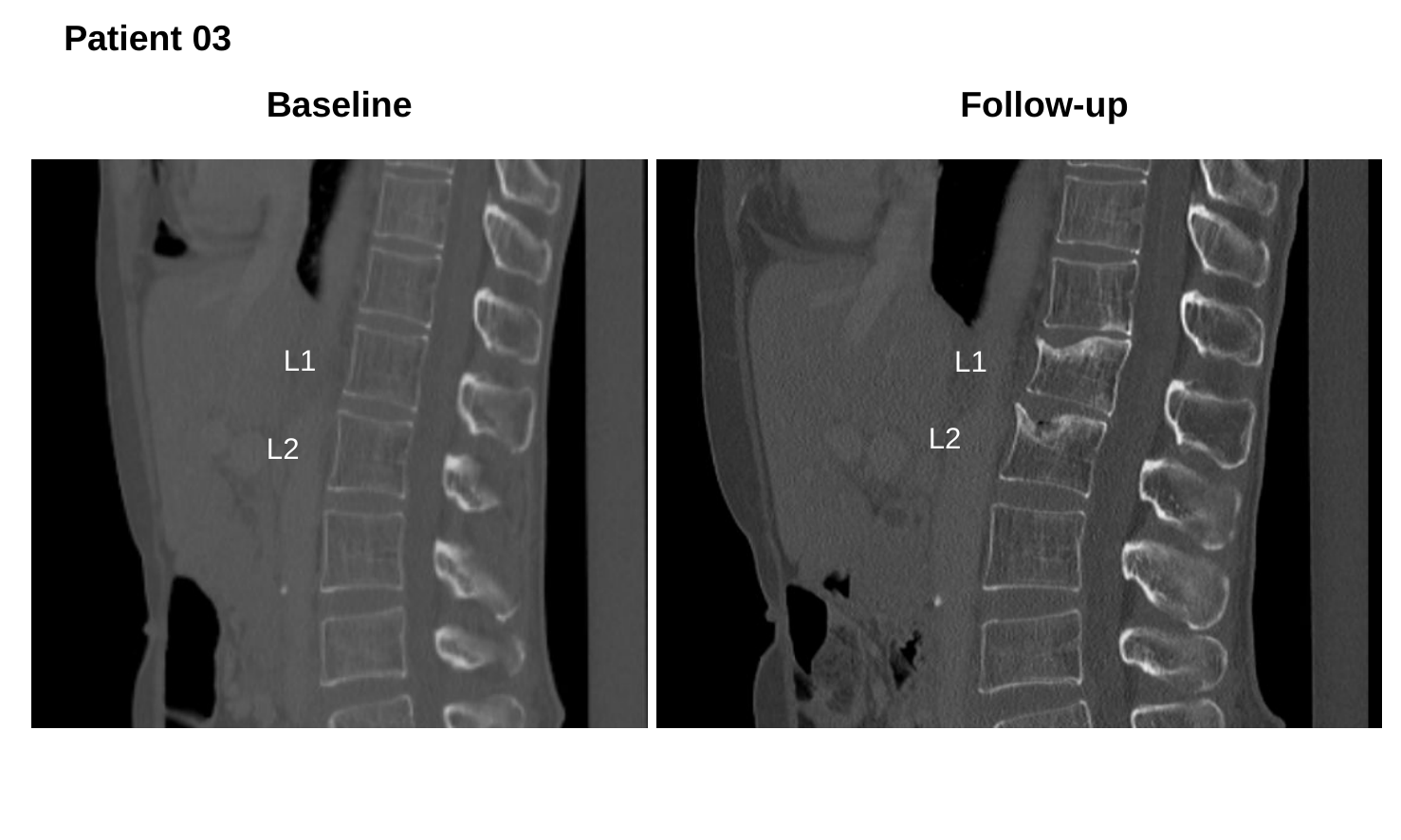

Patient 03
Baseline
Follow-up
L1
L2
L1
L2

## Slide 6
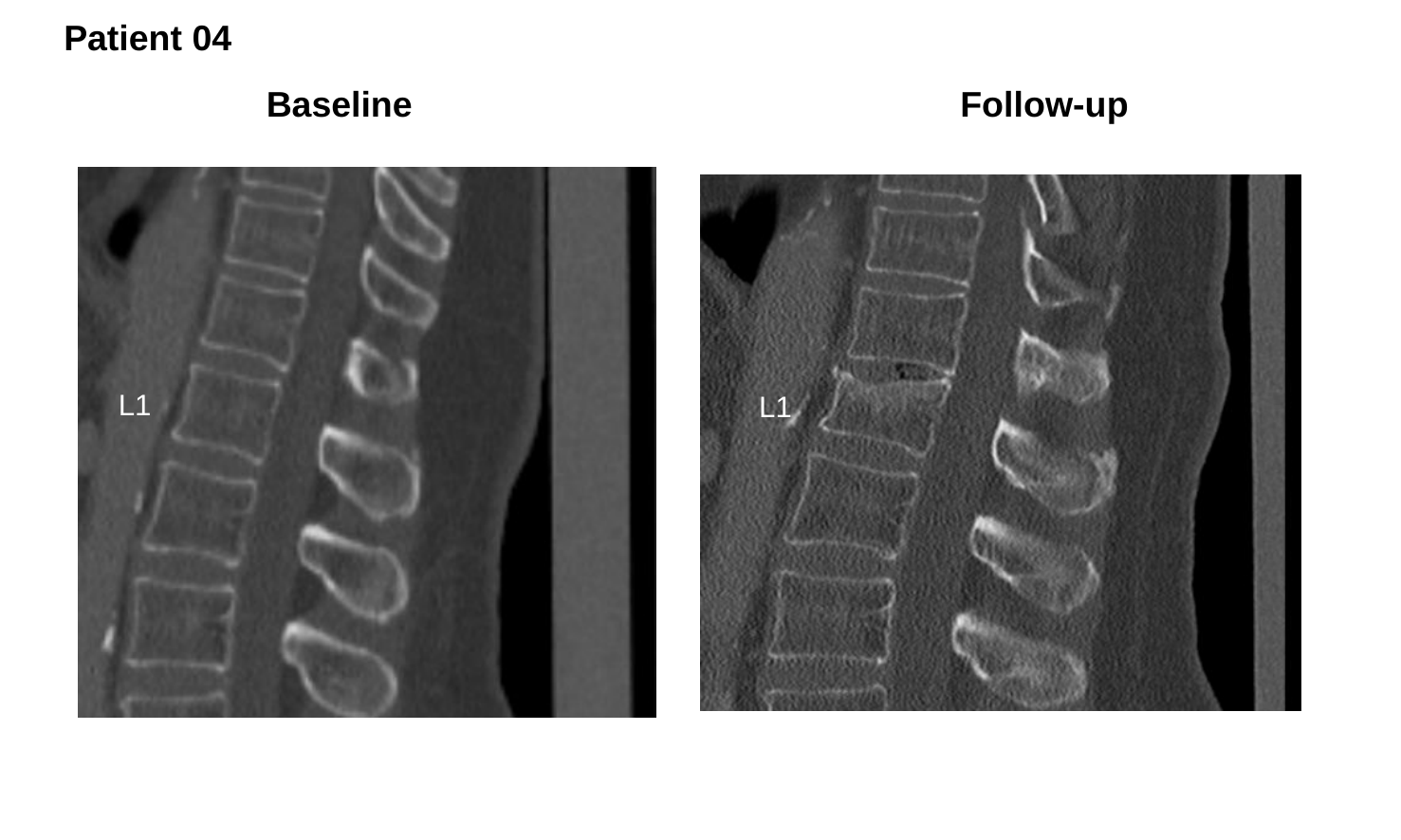

Patient 04
Baseline
Follow-up
L1
L1

## Slide 7
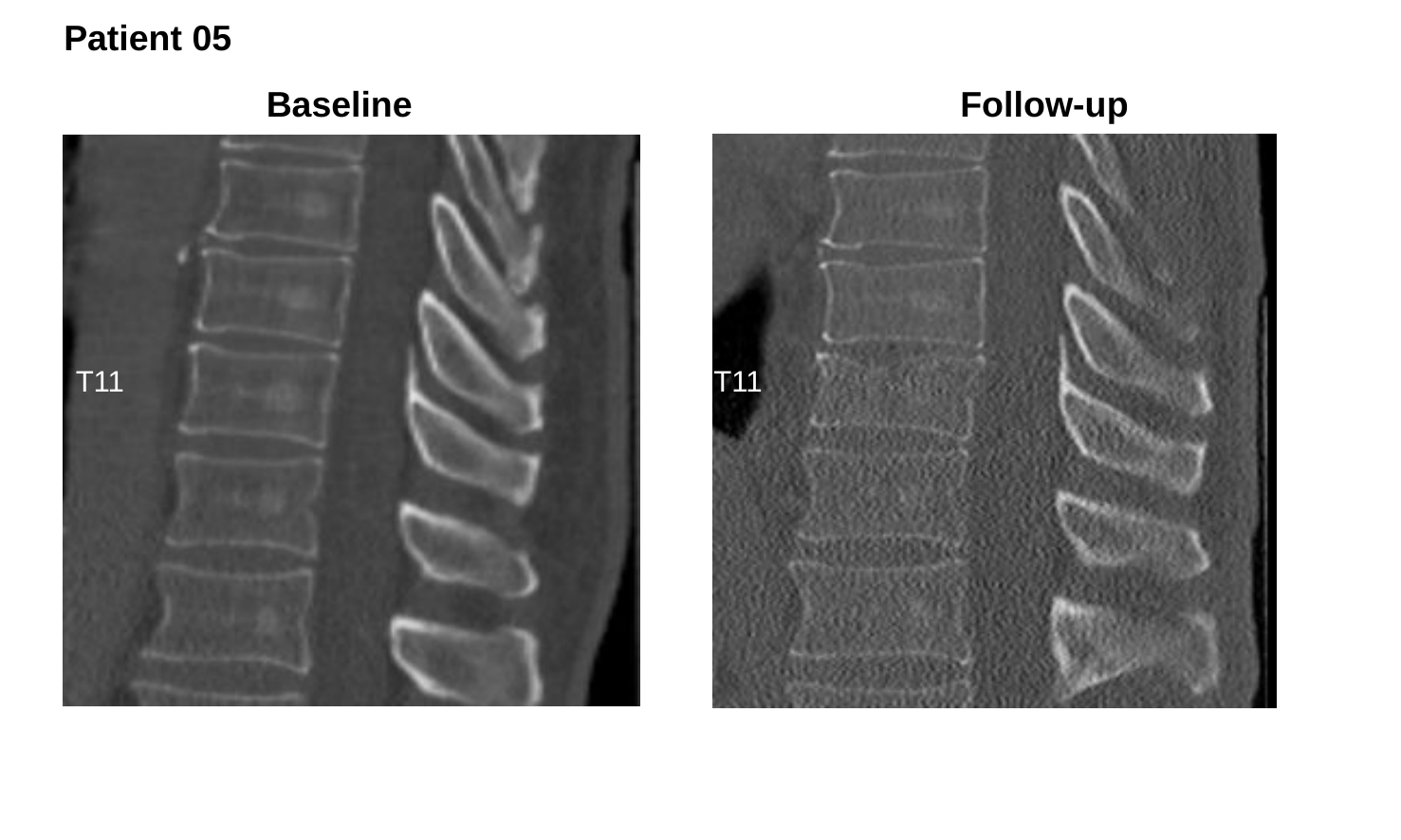

Patient 05
Baseline
Follow-up
T11
T11

## Slide 8
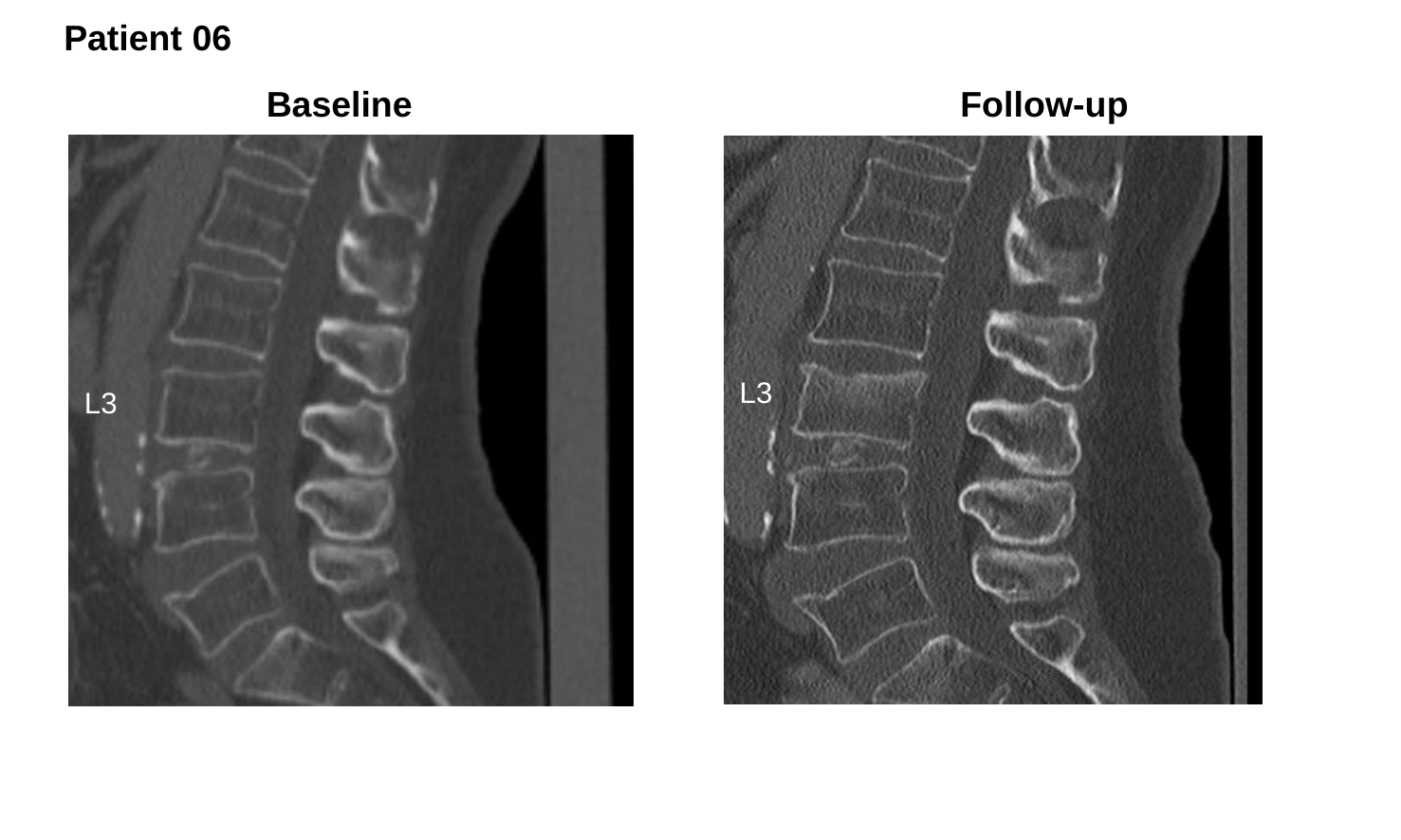

Patient 06
Baseline
Follow-up
L3
L3

## Slide 9
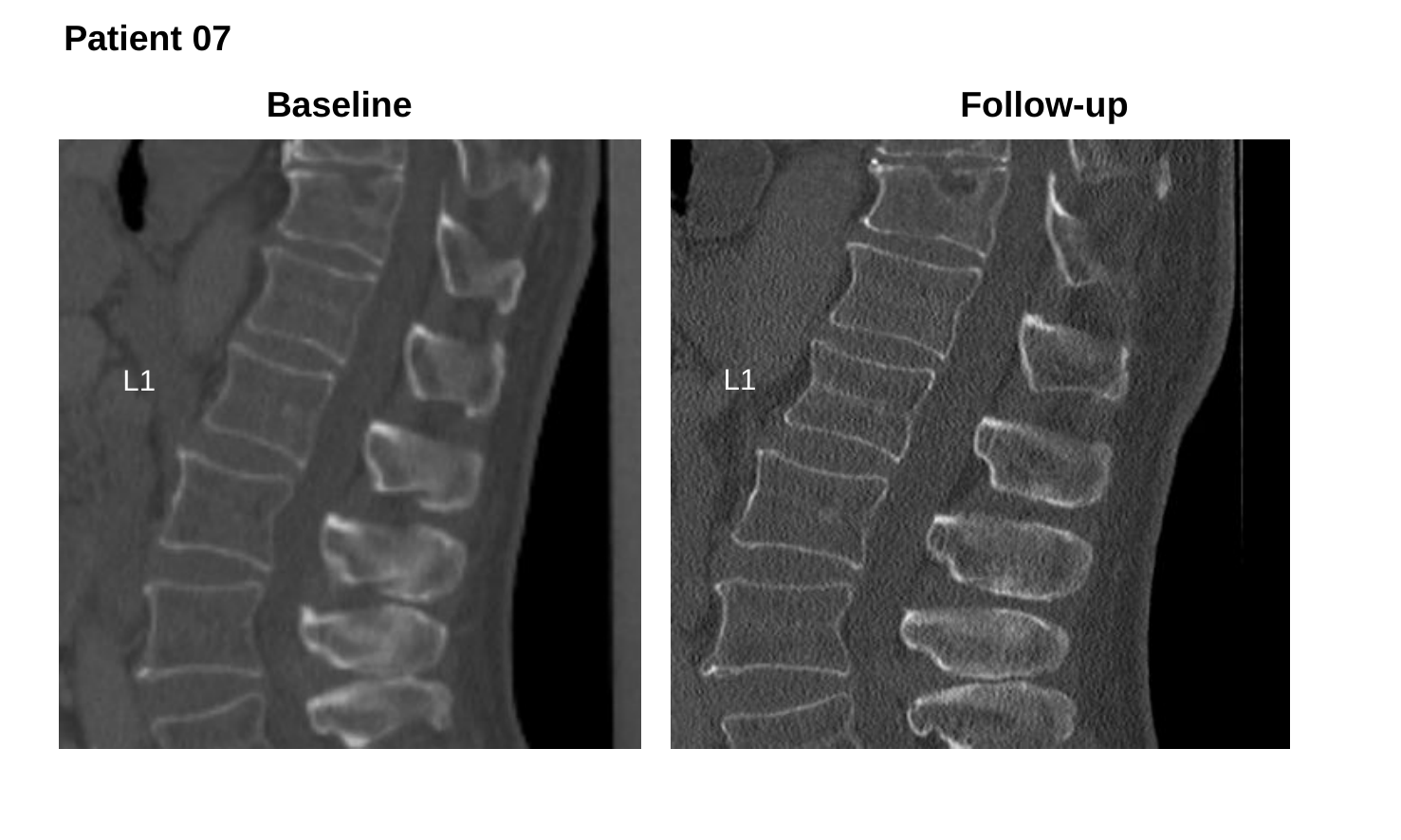

Patient 07
Baseline
Follow-up
L1
L1

## Slide 10
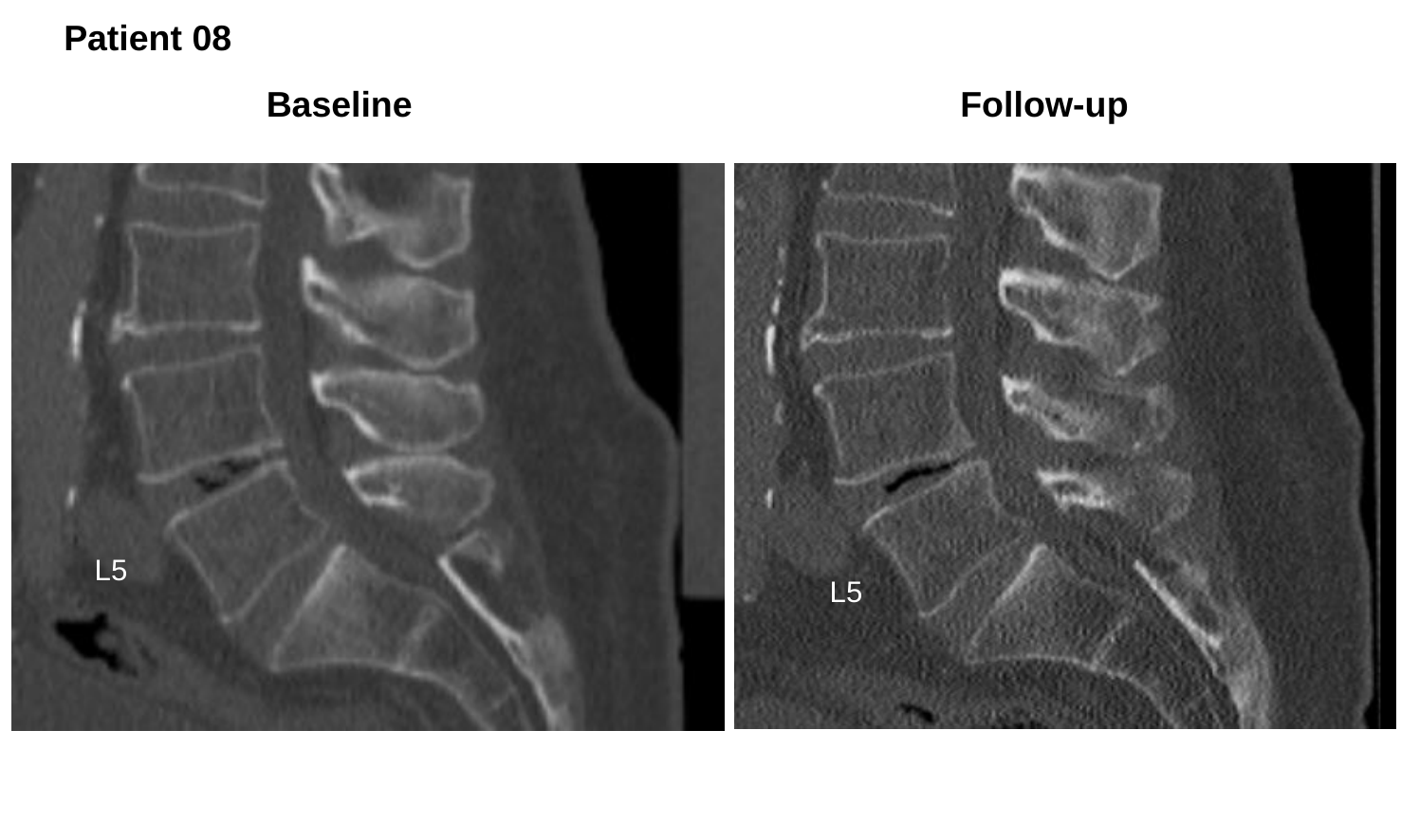

Patient 08
Baseline
Follow-up
L5
L5

## Slide 11
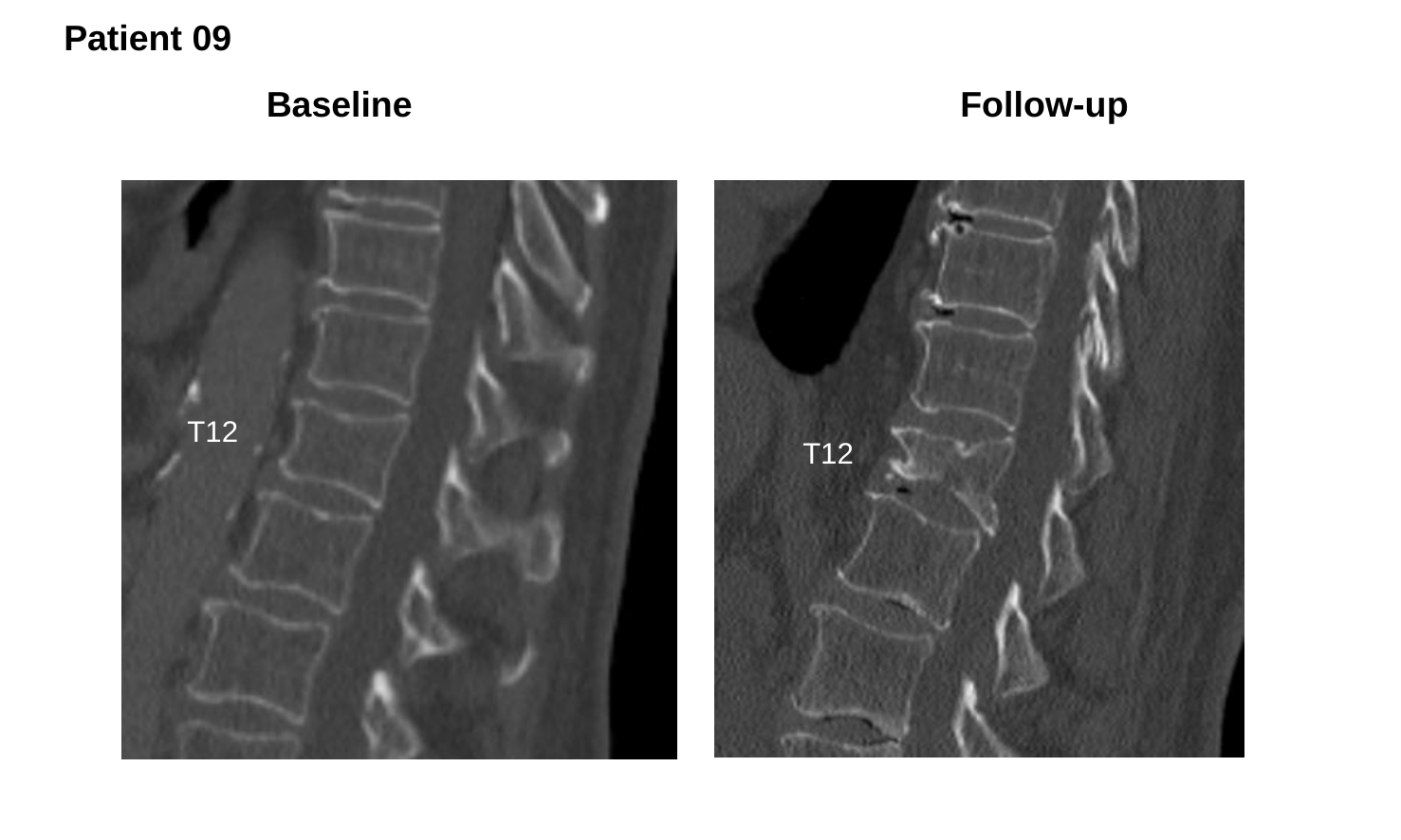

Patient 09
Baseline
Follow-up
T12
T12

## Slide 12
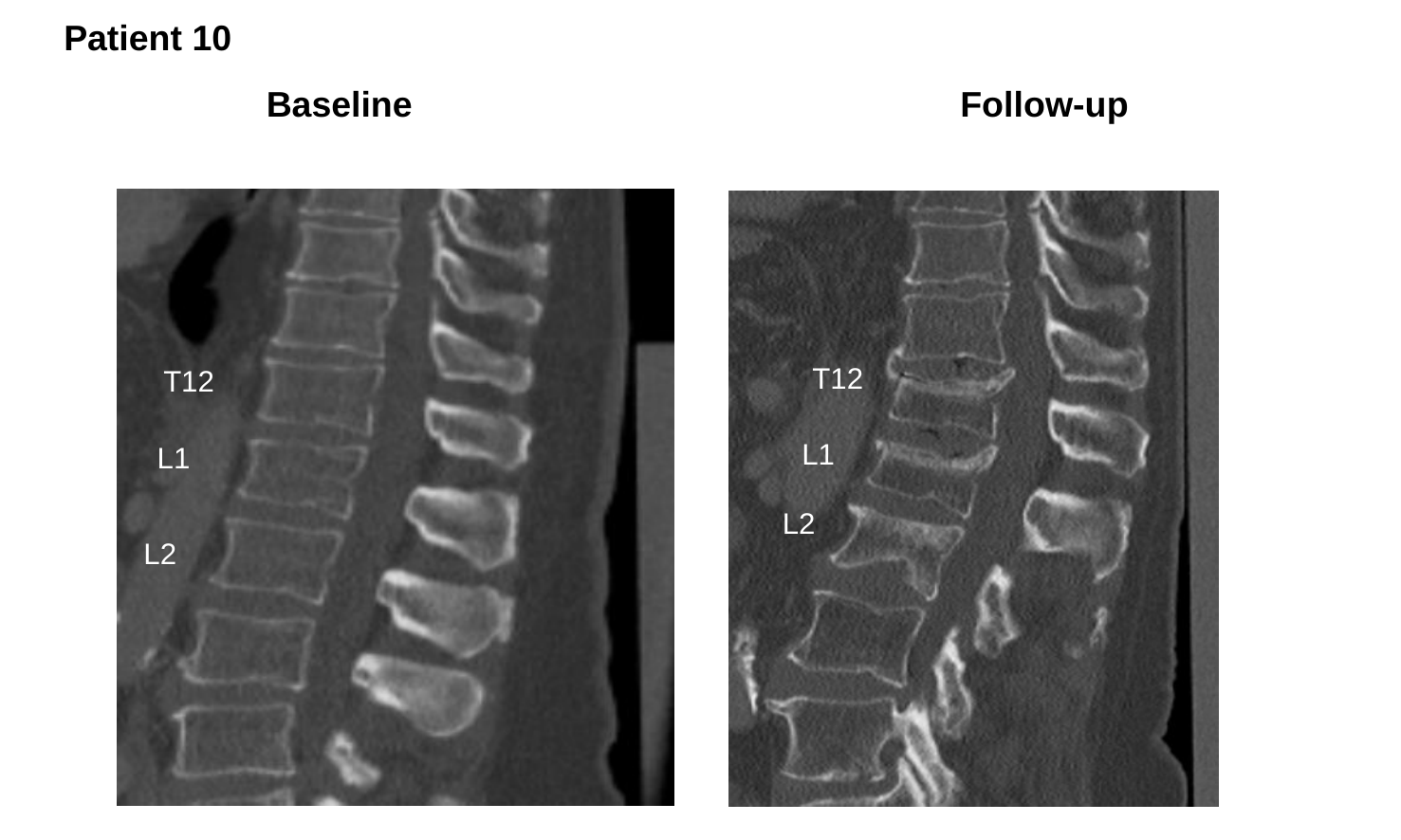

229
Patient 10
Baseline
Follow-up
T12
L1
L2
T12
L1
L2

## Slide 13
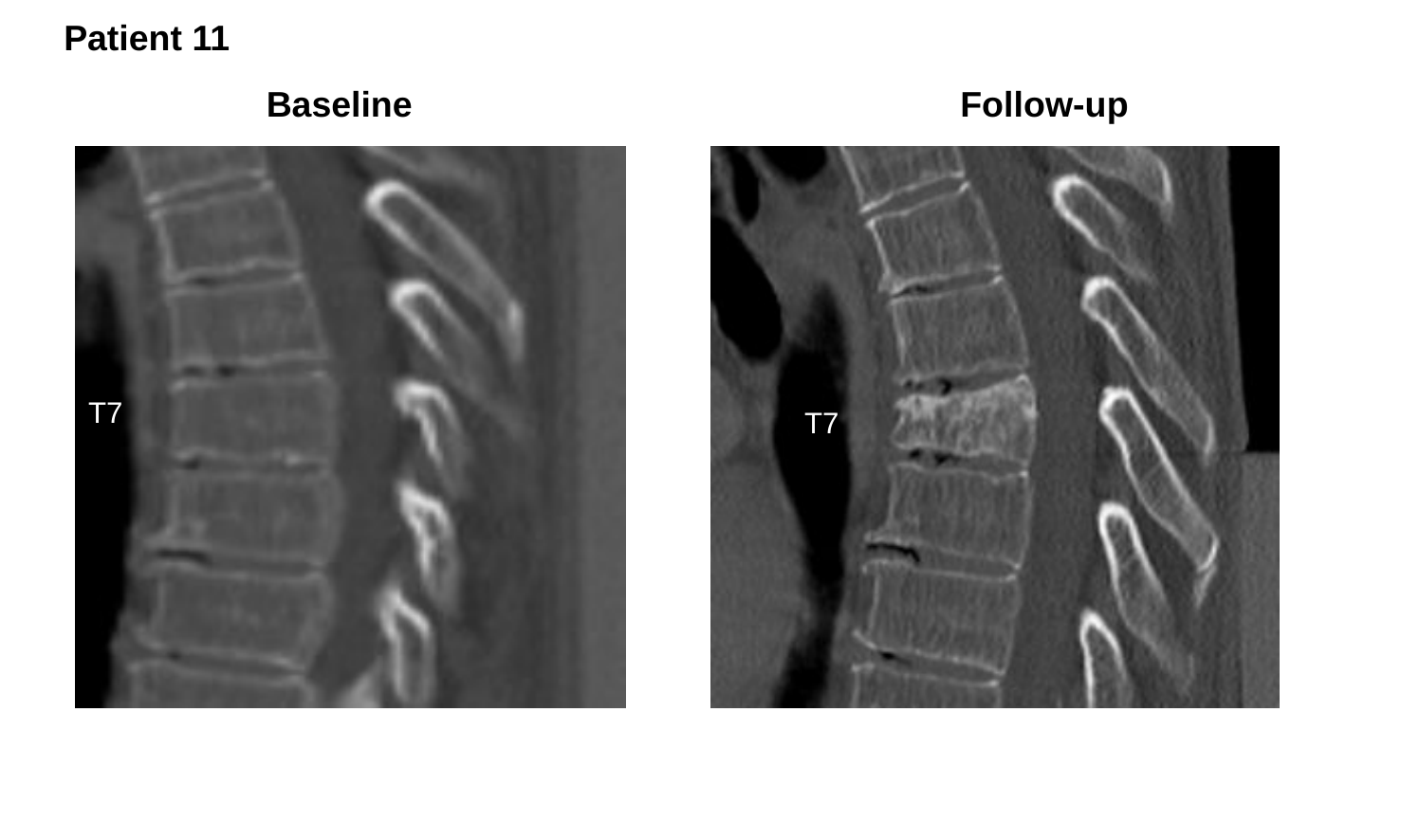

Patient 11
Baseline
Follow-up
T7
T7

## Slide 14
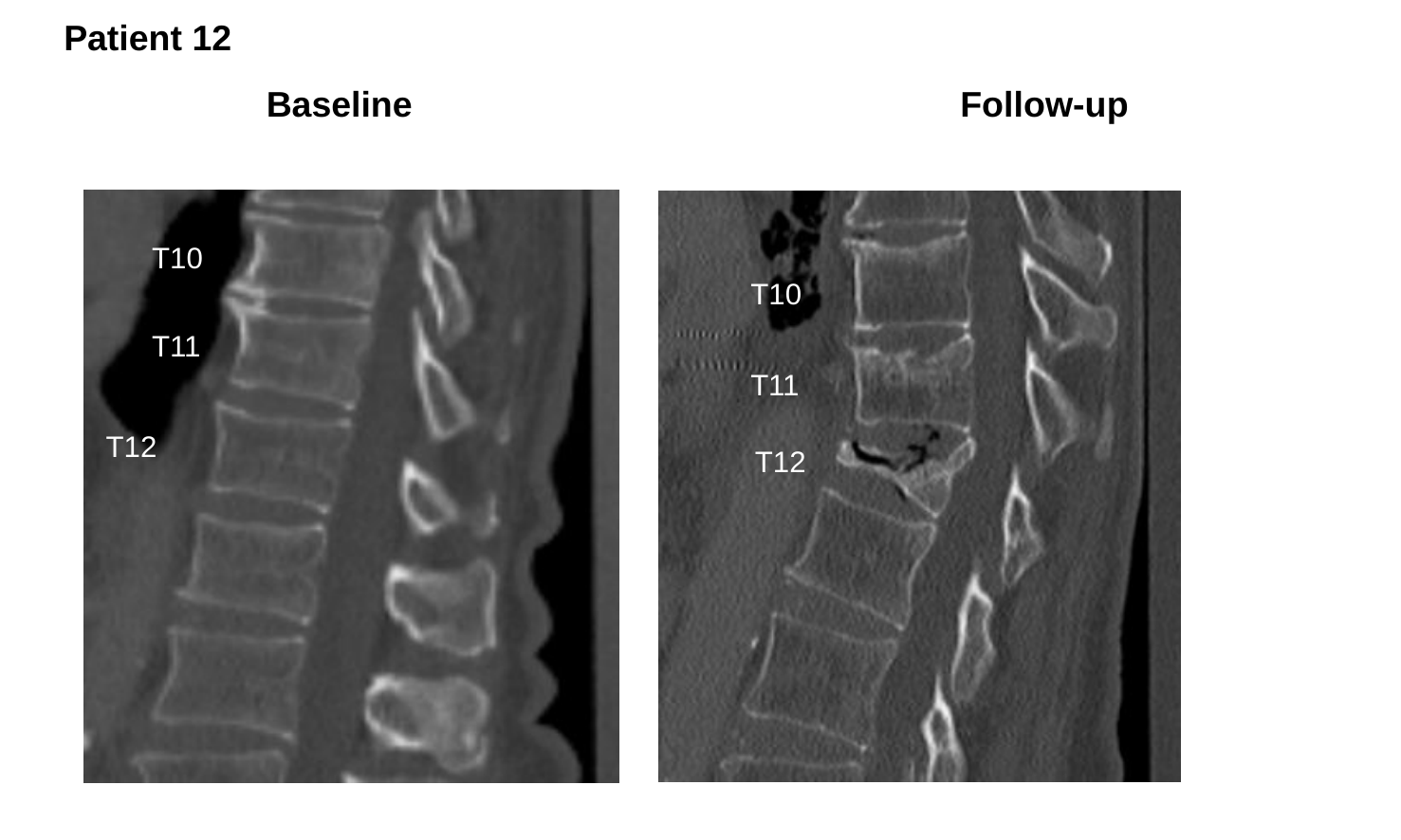

Patient 12
Baseline
Follow-up
T12
T12
T10
T10
T11
T11

## Slide 15
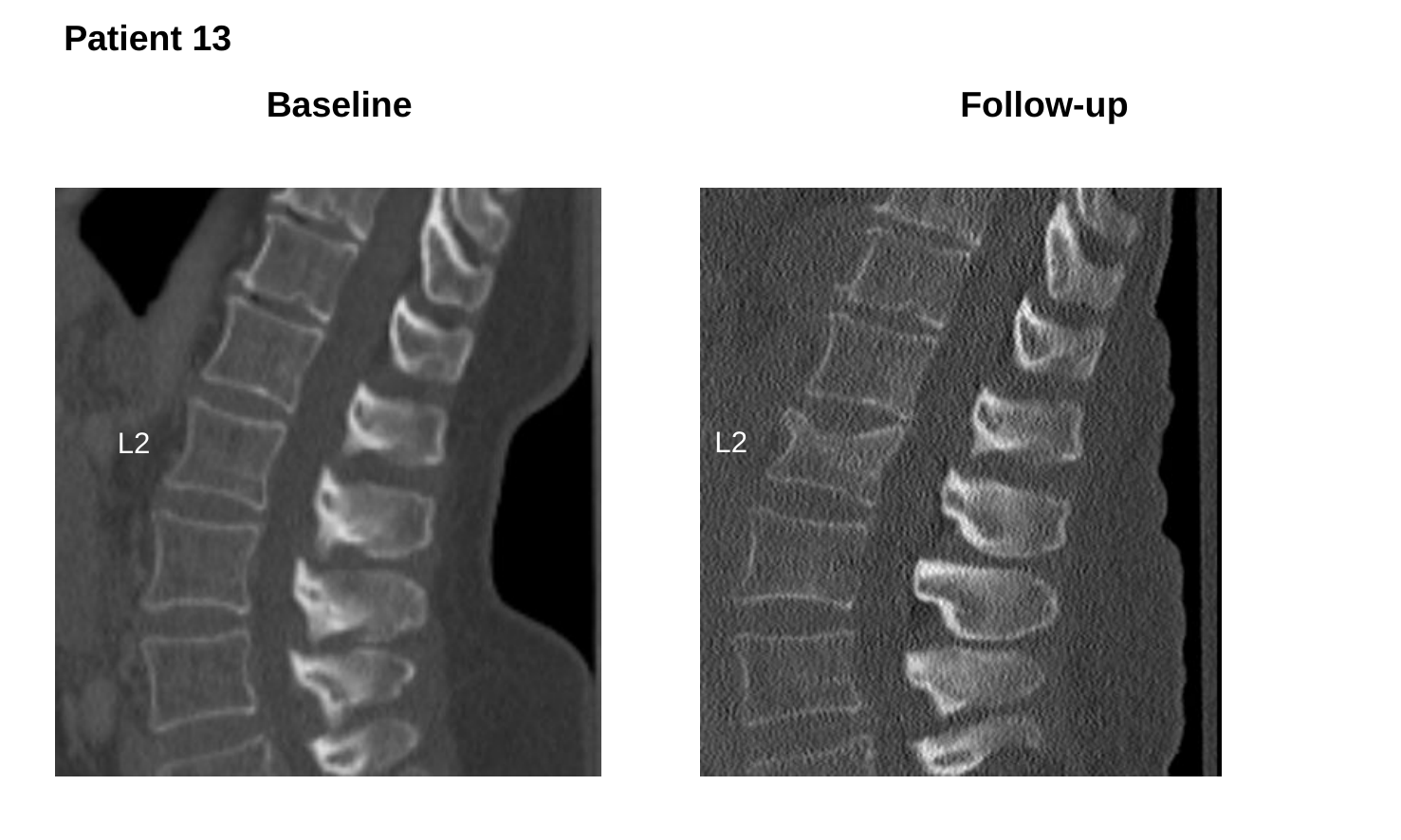

Patient 13
Baseline
Follow-up
L2
L2

## Slide 16
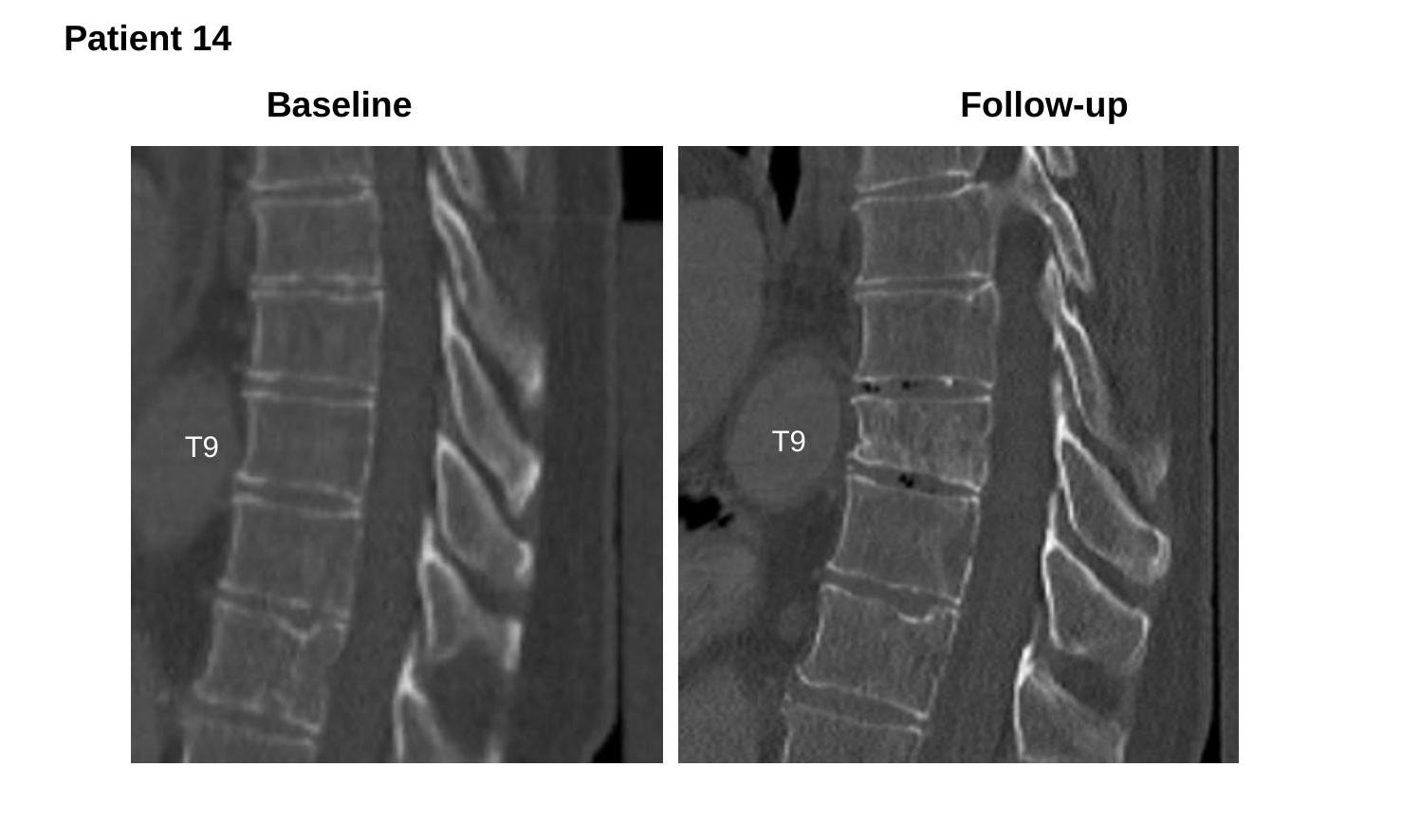

Patient 14
Baseline
Follow-up
T9
T9

## Slide 17
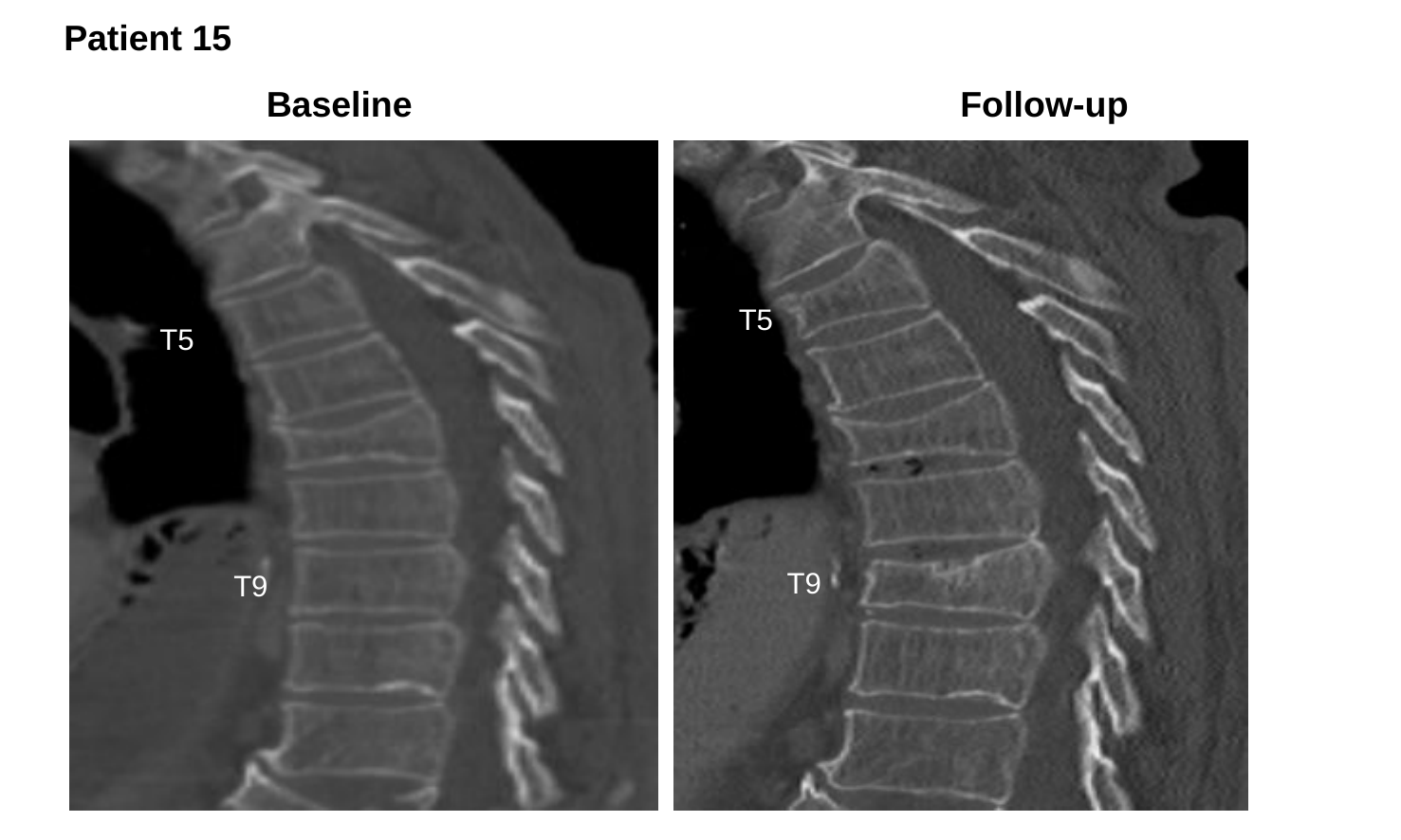

Patient 15
Baseline
Follow-up
T5
T9
T5
T9
T9

## Slide 18
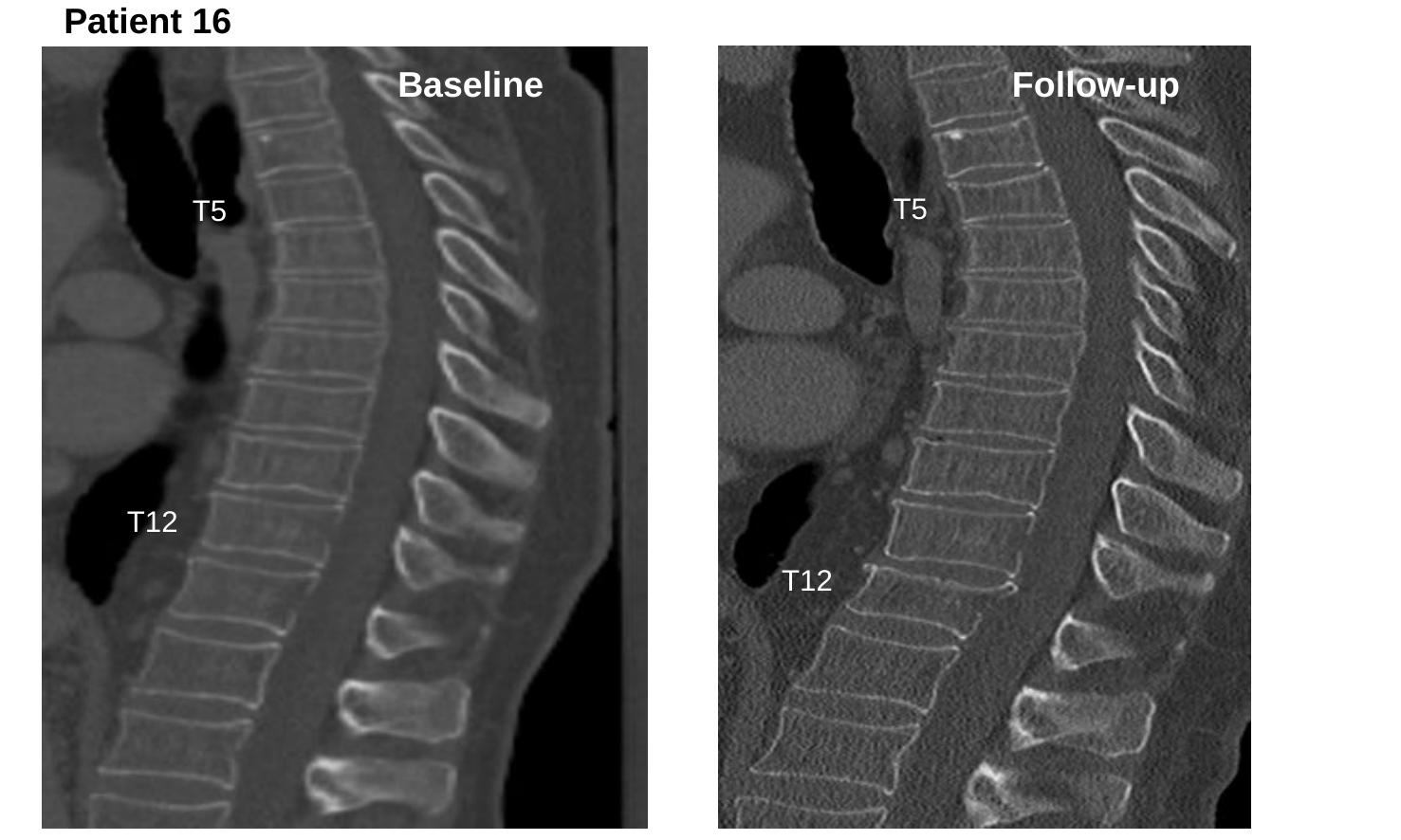

Patient 16
T5
T12
T5
T12
Baseline
Follow-up
